# Severe COVID‐19 pneumonia in an intensive care setting and comparisons with historic severe viral pneumonia due to other viruses

**DOI:** 10.1111/crj.13482

**Published:** 2022-02-24

**Authors:** Kiran Dadhwal, Rosalind Stonham, Hannah Breen, Stephen Poole, Kordo Saeed, Ahilanandan Dushianthan

**Affiliations:** ^1^ General Intensive Care Unit University Hospital Southampton NHS Foundation Trust Southampton UK; ^2^ Department of Microbiology University Hospital Southampton NHS Foundation Trust Southampton UK; ^3^ Faculty of Medicine, University Hospital Southampton University of Southampton Southampton UK; ^4^ NIHR Southampton Clinical Research Facility and NIHR Southampton Biomedical Research Centre, University Hospital Southampton University of Southampton Southampton UK

**Keywords:** COVID‐19, intensive care, viral pneumonia

## Abstract

**Purpose:**

Severe viral pneumonia is associated with significant morbidity and mortality. Recent COVID‐19 pandemic continues to impose significant health burden worldwide, and individual pandemic waves often lead to a large surge in the intensive care unit (ICU) admissions for respiratory support. Comparisons of severe SARS‐CoV‐2 pneumonia with other seasonal and nonseasonal severe viral infections are rarely studied in an intensive care setting.

**Methods:**

A retrospective cohort study comparing patients admitted to ICU with COVID‐19 between March and June 2020 and those with viral pneumonias between January and December 2019. We compared patient specific demographic variables, duration of illness, ICU organ supportive measures and outcomes between both groups.

**Results:**

Analysis of 93 COVID‐19 (Group 1) and 52 other viral pneumonia patients (Group 2) showed an increased proportion of obesity (42% vs. 23%, *p* = 0.02), non‐White ethnicities (41% vs. 6%, *p* < 0.001) and diabetes mellitus (30% vs. 13%, *p* = 0.03) in Group 1, with lower prevalence of chronic obstructive pulmonary disease (COPD)/asthma (16% vs. 34%, *p* = 0.02). In Group 1, the neutrophil to lymphocyte ratio was much lower (6.7 vs. 10, *p* = 0.006), and invasive mechanical ventilation (58% vs. 26%, *p* < 0.001) was more common. Length of ICU (8 vs. 4, *p* < 0.001) and hospital stay (22 vs. 11, *p* < 0.001) was prolonged in Group 1, with no significant difference in mortality. Influenza A and rhinovirus were the most common pathogens in Group 2 (26% each).

**Conclusions:**

Key differences were identified within demographics (obesity, ethnicity, age, ICU scores, comorbidities) and organ support. Despite these variations, there were no significant differences in mortality between both groups. Further studies with larger sample sizes would allow for further assessment of clinical parameters in these patients.

## 1 INTRODUCTION

Severe acute respiratory syndrome coronavirus‐2 (SARS‐CoV‐2) was first identified as a novel human pathogen following reports of pneumonia of unknown aetiology in Wuhan, China, in December 2019. The evolving pandemic has caused greater than three million reported deaths worldwide so far and imposes a significant burden on critical care services, necessitating vast critical care expansion in the United Kingdom, equivalent to 141 new intensive care units (ICUs) over the past 12 months.^1^

Critical illness of COVID‐19 pneumonia is associated with acute hypoxic respiratory failure (AHRF), and for mechanically ventilated patients, the mortality remains high. (These data arise from the Intensive Care National Audit & Research Centre [ICNARC] Case Mix Programme Database. The Case Mix Programme is the national clinical audit of patient outcomes from adult critical care coordinated by the ICNARC. For more information for the representativeness and quality of these data, please contact ICNARC.^2^) The severity of illness is associated with a selective susceptibility of patients with advanced age and cardiovascular and metabolic comorbidities.^3^ Viral pneumonia in general is relatively common and is an important cause of severe respiratory infections in an ICU setting. Reported incidence of severe viral pneumonia varies seasonally worldwide, and the common viruses that cause severe respiratory failure include influenza viruses, respiratory syncytial virus (RSV) and rhinovirus causing significant mortality and morbidity across all age groups.^4^ Although there are several studies from various clinical settings comparing the demographic data, laboratory findings and clinical outcomes of COVID‐19 pneumonia with influenza, there remains lack of comparative studies in the critical care setting. The purpose of this study is therefore to compare the patient specific variables, organ supportive measures and outcomes of patients admitted with COVID‐19 pneumonia with other causes of viral pneumonia of critically ill patients in the intensive care setting.

## 2 METHODS

### 2.1 Study design and participants

We performed a cohort study of adults (>18 years) admitted to the ICU at University Hospital Southampton, UK, who tested positive for respiratory viruses between January 2019 and 2020. Southampton General is a large tertiary referral centre on the south coast of England, offering secondary care services to a population of around 650 000 people.

SARS‐CoV‐2 positive patients were identified retrospectively from laboratory RT‐PCR results between 3 March 2020 and 8 June 2020 who had results corresponding to an ICU admission. Testing was performed on site, initially using the Public Health England (PHE) RpRp‐gene assay and later using the PHE RdRp‐gene and E‐gene combined assay. Respiratory virus testing at this time was restricted to SARS‐CoV‐2 alone due to limited availability of reagent during the first peak of the pandemic.

The comparator group were identified in the same way, with a positive laboratory PCR result between 1 January 2019 and 31 December 2019. The laboratory assay tested for adenovirus, influenza A, influenza B, human metapneumovirus (hMPV), parainfluenza viruses (PIV) (Types 1–3), human rhinovirus (hRV) and RSV. The study was approved by local ethics committee for retrospective data collection (RHM CRI 0370/IRAS 232922), and due to the nature of the study, patient consent was waived. This study is reported according to the STROBE guideline.^5^

### 2.2 Data collection

Anonymised data were collected from existing electronic hospital patient records. All relevant clinical details were collected including baseline patient demographics, comorbidities (described using Charlson's comorbidity index^6^), Acute Physiology and Chronic Health Evaluation II (APACHE II) score, the Sequential Organ Failure Assessment (SOFA) score, oxygenation index (PaO_2_/FiO_2_), admission laboratory variables and events during GICU admission including use of non‐invasive ventilation (NIV), the need for mechanical ventilation and haemofiltration. The outcomes reported are duration of ICU length of stay, total hospitalised days, 28‐day mortality, ICU mortality and hospital mortality. APACHE II variables were collected at the first 24 h of ICU admission. The oxygenation index represents lowest PaO_2_/FiO_2_ on admission to ICU. Severe community‐acquired pneumonia is defined in accordance to published American Thoracic Society/Infectious Diseases Society of America guidelines.^7^

### 2.3 Statistical analysis

Baselines characteristics of all positive viral detections were summarised using appropriate descriptive statistics. Continuous data are presented as medians and interquartile ranges (IQRs) and categorical data as numbers and percentages. Absolute differences between proportions are presented with 95% confidence intervals. Differences and 95% confidence intervals between medians were calculated using the Hodges–Lehmann estimator. Comparative statistical tests were performed between SARS‐CoV‐2 and non‐SARS‐CoV‐2 viral positive cases. For descriptive statistics, data are presented as median with IQRs as variables were found to be non‐normal distribution assessed by the Kolmogorov–Smirnov test. Comparison of proportions was performed using two‐sided Fisher's exact test. Data analysis was performed using GraphPad Prism 8.4 (GraphPad Software, LLC).

## 3 RESULTS

There were 93 COVID‐19 patients admitted to the ICU during March 2020 to June 2020 for respiratory support and 52 non‐SARS‐CoV‐2 viral pneumonia patients admitted in the year 2019 who met the criteria for the inclusion in the study. We classified the COVID‐19 pneumonia patients as Group 1 and the historical cohort as Group 2. The demographics for both groups are presented in Table 1. The median age for the COVID‐19 group was much lower at 57 (IQR 47, 65). Moreover, although there was an increased male predominance for COVID‐19, this was not statistically significant. The median onset of symptoms was shorter for non‐COVID‐19 viral pneumonia. There was an increased proportion of obesity with BMI > 30 kg/m^2^ in the COVID‐19 group accounting for 43% in comparison with less than quarter in Group 2. There was an increased ethnicity prevalence in COVID‐19 group, and 37% of all admissions were non‐White Caucasian (Table 1).

**TABLE 1:** Patient demographics on admission, disease severity indices, intensive care interventions and admission laboratory markers from all COVID‐19 admitted patients

Variables	Group 1 COVID 19 pneumonia (*N* = 93)	Group 2 Other viral pneumonia (*N* = 53)	*p* value
Age	58 (47, 66)	65 (50, 71)	0.13
Male (%)	60%	45%	0.09
Symptomatic days prior to admission	7 (5, 10)	4 (3, 7)	<0.001^*^
BMI > 30 kg/m^2^, *n* (%)	39 (41.9%)	12 (22.6%)	0.02^*^
Clinical Frailty Scale (CFS)	2 (1, 3)	3 (3, 5)	<0.001^*^
Charlson's comorbidity index (CCI)	2 (1, 3)	3 (1.5, 5)	0.02^*^
Race/ethnic group			
White, *n* (%)	55 (59%)	50 (94%)	<0.001^*^
Other, *n* (%)	38 (41%)	3 (6%)	
Comorbidities, *n* (%)			
COPD/asthma	15 (16.1%)	18 (34.0%)	0.02^*^
Other respiratory disease	7 (7.5%)	7 (13.2%)	0.38
Chronic kidney disease	7 (7.5%)	7 (13.2%)	0.38
Congestive cardiac failure	4 (4.3%)	3 (5.7%)	0.70
Diabetes mellitus	28 (30.0%)	7 (13.2%)	0.03^*^
Hypertension	38 (40.9%)	14 (26.4%)	0.11
Ischaemic heart disease	8 (8.6%)	5 (9.4%)	0.99
Immunosuppression	13 (14.0%)	13 (24.5%)	0.12
ICU severity indices			
APACHE II score	14 (11, 23)	14 (9.5, 19)	0.18
SOFA score	4 (3, 6)	3 (2, 6)	0.09
PaO_2_/FiO_2_ ratio (mmHg)	114.8 (99.0, 137.3)	126.8 (95.3, 203.3)	0.11
Admission laboratory profiles			
Bilirubin (mmol/L)	11 (8, 16)	10 (7, 10)	0.22
Creatinine (mmol/L)	72 (57, 101)	75 (60, 107)	0.31
C‐reactive protein (mg/L)	154 (102, 209)	133 (60, 204)	0.31
Lymphocytes 10^9^/L	0.8 (0.6, 1.3)	0.8 (0.5, 1.3)	0.31
Neutrophil/lymphocyte ratio	6.7 (5, 12.2)	10.0 (7.7, 19.5)	0.006^*^
White cell counts 10^9^/L	8.1 (5.6, 11.7)	10.7 (7.1, 16.0)	0.004^*^
ICU organ support			
Cardiovascular support, *n* (%)	30 (32.2%)	16 (30.2%)	0.85
Non‐invasive ventilation alone, *n* (%)	35 (37.6%)	30 (56.6%)	0.04^*^
Mechanical ventilation, *n* (%)	54 (58.1%)	14 (26.4%)	<0.001^*^
Renal replacement therapy, *n* (%)	19 (20.4%)	5 (9.4%)	0.11

*Note*: Data are presented as median (interquartile range) or numbers (percentage) unless otherwise stated.

Abbreviations: APACHE II, Acute Physiology and Chronic Health Evaluation II; BMI, body mass index; COPD, chronic obstructive pulmonary disease; PaO_2_/FiO_2_, ratio of arterial oxygen partial pressure to fractional inspired oxygen; SOFA, Sequential Organ Failure Assessment.

^*^ *p* < 0.05 as assessed by Fisher's exact test for categorical variables and Mann–Whitney *U* test for continuous variables.

Charlson's comorbidity index was higher in patients with non‐COVID‐19 viral pneumonia group with a proportional variation in individual comorbidities between both groups. There was an increased prevalence of respiratory comorbidities, particularly airway disease with asthma and chronic obstructive pulmonary disease (COPD) among Group 2, whereas diabetes mellitus was more frequent in COVID‐19 pneumonia. The ICU admission severity indices (APACHE II and SOFA) were similar between groups with a slightly lower worse admission oxygenation defined by PaO_2_/FiO_2_ ratio in the COVID‐19 group which was not statistically significant. The estimated in‐hospital mortality from the calculated APACHE II scores at presentation for both groups was around 15%. The baseline routine laboratory variables such as bilirubin, creatinine, C‐reactive protein (CRP), lymphocytes and white cell counts did not show any difference between both groups. However, the neutrophil to lymphocyte ratio was higher in patients with non‐COVID pneumonia (Table 1).

**FIGURE 1:**
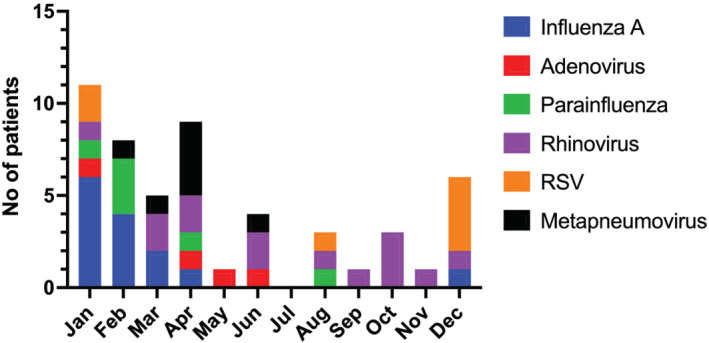
Seasonal variations of severe viral pneumonia ICU admissions for the year between January–December 2019

Among the patients with non‐COVID‐19 viral pneumonia (Group 2), the viral pathogens identified were influenza A (26.4%), rhinovirus (26.4%), RSV (15.1%), metapneumovirus (13.2%), parainfluenza T3 (11.3%) and adenovirus (7.8%) (Table 2). As expected, there was an increased seasonal prevalence of influenza and RSV viruses during winter months, and rhinovirus did not show seasonal variation (Figure 1). Cobacterial infection defined as positive bacterial microbiology during the first 48 h of ICU admission was 16.4% and 11.3%, respectively. Moreover, there were differences in the type of bacterial infection between both groups (Table 3).

**TABLE 2:** Viral pathogens identified in Group 2 and the seasonality of their infections

Type of virus	Number (%) (*N* = 53)
Influenza A	14 (26.4%)
Rhinovirus	14 (26.4%)
Respiratory syncytial virus	8 (15.1%)
Metapneumovirus	7 (13.2%)
Parainfluenza T3	6 (11.3%)
Adenovirus	4 (7.5%)

**TABLE 3:** Coinfections identified during ICU admission

Pathogen	Viral pneumonia *N* = 53	COVID‐19 pneumonia *N* = 93
*Staphylococcus aureus*	1	3
*Streptococcus pneumoniae*	2	‐
Pneumococcus	2	‐
*Pseudomonas aeruginosa*	1	‐
Klebsiella	‐	1
*Proteus mirabilis*	‐	1
*Escherichia coli*	‐	1
Proportion of patients with significant coinfection identified	11.3%	6.4%

*Note*: Coinfection is defined as significant pathogen identified in 48 h prior to or after admission to critical care.

**FIGURE 2:**
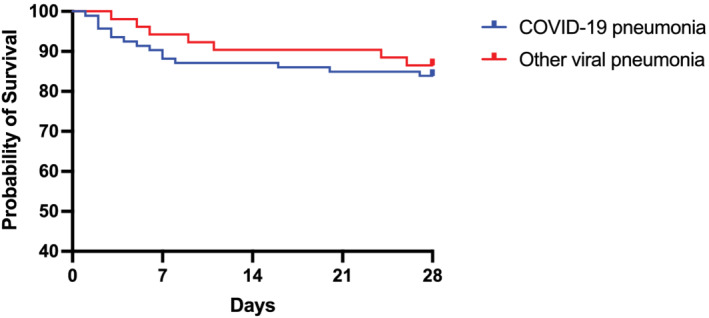
Survival probability at Day 28 between critically ill ICU COVID‐19 pneumonia and other viral pneumonia patients

Although there was no difference in the need for cardiovascular support between groups, invasive mechanical ventilation was more common in COVID‐19 pneumonia (58% Group 1 vs. 26% Group 2), whereas more patients in Group 2 had NIV alone to manage their respiratory failure. The incidence of renal replacement therapy was also much higher in COVID‐19 patients (20% Group 1 vs. 9% Group 2). There were no differences in 28 days, ICU or hospital mortality between two groups (Table 4 and Figure 2). The overall hospital mortality was 18% and 22.6% for Groups 1 and 2, respectively. However, the median length of ICU and hospital stay was much more prolonged for COVID‐19 patients.

**TABLE 4:** Outcome of all patients

Outcomes	Group 1 COVID 19 pneumonia (*N* = 93)	Group 2 Other viral pneumonia (*N* = 53)	*p* value
Length of ICU stay (days)	8 (3, 22)	4 (2, 7)	<0.001^*^
Length of hospital stay (days)	22 (10, 36)	11 (7, 24)	<0.001^*^
28‐day mortality, *n* (%)	15 (16.1%)	8 (15.1%)	0.99
ICU mortality, *n* (%)	16 (17.2%)	11 (20.7%)	0.66
Hospital mortality, *n* (%)	16 (17.2%)	12 (22.6%)	0.51

*Note*: Data are presented as median (interquartile range) or numbers (percentage) unless otherwise stated.

^*^ *p* < 0.05 as assessed by Fisher's exact test for categorical variables and Mann–Whitney *U* test for continuous variables.

## 4 DISCUSSION

This study compared the differences in demographics, organ support measures and clinical outcomes during ICU admissions of severe COVID‐19 pneumonia with historic patients admitted with other viral pneumonia from the previous year. The COVID‐19 group was less frail as defined by the clinical frailty score (CFS) and had quantitatively lower overall Charlson's comorbidity index score. Although the COVID‐19 ICU patients were younger by a median age of 8 years (median age of 58 vs. 65), this was not statistically significant. Moreover, there was a trend towards increased male predominance (60% vs. 45%) in the COVID‐19 group. Obesity (defined by a body mass index [BMI] of >30) and diabetes mellitus were more prevalent in COVID‐19 pneumonia. Likewise, non‐White ethnicity is proportionally higher in COVID‐19 pneumonia (41% vs. 6%). Airway diseases with asthma and COPD were more common in non‐COVID‐19 viral pneumonia. Similar to other published cohort studies, influenza A and rhinovirus were the most common causes of severe respiratory failure leading to ICU admission accounting for >50% of viral pneumonia in our population.^4^, ^8^, ^9^ The seasonality of severe viral infection was as anticipated, where influenza and RSV were more prevalent in winter months. Rhinovirus was present throughout the year.^4^

Despite the similarities in the ICU severity indices such as APACHE II, SOFA and oxygenation index defied as PaO_2_/FiO_2_ (mmHg) ratios between groups, there were variations in ICU organ support measures provided. More patients with COVID‐19 needed invasive mechanical ventilation and a trend towards increased renal replacement therapy requirement, which was not significant. The onset of symptoms to hospitalisation is prolonged in COVID‐19 patients. This is consistent with already established timelines of COVID‐19 reaching peak severity at 7–10 days and suggests that other viral pneumonia has a more acute onset of peak severity.^10^ Moreover, COVID‐19 patients also had a higher median number of total hospital days, consistent with a study from Finland showing longer hospitalisation in COVID‐19 patients compared to those with influenza.^11^ Regardless of these differences, the ICU, hospital and 28‐day mortalities were similar between both groups. Our finding of similar mortality is consistent with a large multicentre study comparing critically ill COVID‐19 with H1N1 influenza pandemic during 2009.^12^ Our overall mortality is lower than previously published national and international data sets.^2^, ^13^ In contrast, an American study comparing critically ill hospitalised patients with influenza and COVID‐19 showed a mortality of 40% in COVID‐19 group with a significantly lower mortality among the influenza group.^14^ However, our critically ill cohort was inclusive of all respiratory viruses, and the variations in mortality could be accounted for by local practice and management guidelines, as our study only consisted of analysis of single‐centre data.

Similar to our findings, one prospective study found that those with COVID‐19 were less likely to have underlying pulmonary disease.^11^ Our findings are also consistent with regard to patient's characteristics including the type of comorbidities, and others have also highlighted that those hospitalised or critically ill COVID‐19 patients are more likely to have obesity, diabetes mellitus and kidney disease or be of Black race than those with influenza.^12^, ^14^ Moreover, those hospitalised with COVID‐19 appears to have increased tendency to develop acute respiratory distress syndrome, pulmonary embolism, septic shock and haemorrhagic stroke than those with influenza but less frequently develop atrial fibrillation or myocardial infarction.^15^

Despite the similar ICU admission scores and the degree of hypoxia at presentation, there was an increased need for mechanical ventilation in the COVID‐19 group with associated prolonged duration of hospitalisation. Although renal replacement therapy was also more common in COVID‐19 patients, this was not statistically significant. This is consistent with previously published data that a higher proportion of patients with COVID‐19 required intensive care and mechanical ventilation than those with influenza, with an increased risk of developing extra pulmonary organ dysfunction.^11^, ^15^, ^16^ In contrast, an Australian study comparing COVID‐19 patients with H1N1 influenza pandemic in 2009 concluded that fewer proportions of COVID‐19 admissions needed invasive mechanical ventilation.^12^ This is likely due to the reflection of variations in pandemic severity and utilisation of ICU healthcare between countries. Although most admission laboratory variables were similar between groups, the neutrophil/lymphocyte ratio (NLR) was significantly lower in the COVID‐19 group. This finding of high NLR in the non‐COVID‐19 group may imply increased neutrophilic response possibly due to associated bacterial coinfections in this group.

Our study has several limitations. This was a single‐centre retrospective cohort study with a small group of patients. Larger samples are needed for further confirmation of study findings. Data from both groups were taken during different time periods—this is attributed to a significant lack in other respiratory viruses presenting to hospital following social distancing and guidance from PHE not to test for other respiratory viruses during the first peak to save reagents for COVID‐19 testing. Moreover, we only started performing an extended blood panel analysis including variables such as D‐dimer, lactate dehydrogenase, ferritin and high‐sensitive cardiac troponin during the COVID‐19 pandemic, and as a result, these were not done routinely or consistently in viral pneumonia patients. Therefore, we were unable to directly compare these extended panels between both groups. However, despite these limitations, our findings highlight the impact of pandemics, in this case, SARS‐CoV‐2, on ICU services and comparing it with the impact of other infections on the same ICU during a nonpandemic status. These could aid in addressing strategic planning in designing future services and ICU provisions for future pandemics, for example, increasing ICU beds.

## 5 CONCLUSION

To our knowledge, this is the first cohort study from the United Kingdom comparing ICU admissions of severe non‐COVID‐19 viral pneumonia with COVID‐19 pneumonia. We have identified key differences in patient specific variables such as onset of symptoms prior to hospitalisation, BMI, frailty score and the type of comorbidities. Proportionately more patients with COVID‐19 patients had obesity, diabetes mellitus and lesser chronic respiratory comorbidities. The COVID‐19 patients also had lower neutrophil to lymphocyte ratio. Despite these differences, the ICU specific scores were similar, and there was no difference in mortality between groups. The COVID‐19 patients had prolonged ICU stay and overall hospitalisations. In comparison with previously published data from international studies, our findings are consistent, except the key difference of lower ICU and hospital mortality in our population.

## CONFLICT OF INTEREST

Nothing to declare or disclose.

## AUTHOR CONTRIBUTIONS

AD and KS conceived and designed the study and contributed to data collection, data analysis and manuscript writing and editing. KD, HB and RS contributed to data collection and manuscript writing and editing. SP contributed to data collection and manuscript editing.

## ETHICS STATEMENT

The study was approved by local ethics committee for retrospective data collection (RHM CRI 0370/IRAS 232922), and due to the nature of the study, patient consent was waived.

## Data Availability

Data are available on request.
